# Implementing health system change: What are the lessons from the African Health Initiative?

**DOI:** 10.1186/1472-6963-13-S2-S14

**Published:** 2013-05-31

**Authors:** Lucy Gilson

**Affiliations:** 1 Health Policy and Systems Division, School of Public Health and Family Medicine, University of Cape Town, South Africa; 2 Department of Global Health and Development, London School of Hygiene and Tropical Medicine, UK

## 

The five African Health Initiative Population Health Implementation and Training (PHIT) Partnerships represent a rich and important set of health system strengthening initiatives. All can be called *whole system* strengthening initiatives in two important respects.

First, from a *health system* perspective, as explicitly discussed, all the PHIT Partnerships are multi-dimensional, seeking to achieve performance improvements by working across the building blocks and levels of the health system. All address resource needs (human, financial, and supplies) in some way. The common focus on strengthening information use in clinical and managerial decision-making, meanwhile, tackles what some regard as the key leverage point for health system improvement [1] and quality of care is another such point [2]. Considering the role of community health workers, moreover, emphasizes that the health system stretches beyond the doors of health facilities, and that health system development requires combined community and facility-based actions. Finally, several initiatives emphasize the importance of strengthening supervision and management coaching and mentoring. Importantly, the different activities within each partnership are intended to work synergistically together.

Second, although less clearly outlined in these papers, the PHIT Partnerships all reflect key features of complex social programs (Table 1) and are, in themselves, dynamic and *complex adaptive systems*[3]. They have all evolved over time, being developed and adapted in response to experience in implementation, for example. They have also all worked through a range of people and relationships. Indeed, as *partnerships*, the very essence of these projects is a relationship between actors outside the health system and those working within it: health workers, facility teams, supervisors, district management teams and so on. Finally, they have been implemented within, affecting and being affected by, dynamic, multi-layered contexts – encompassing histories and past experiences (e.g. of resource availability, management or the usual ways of working), wider sets of actors and agents (including politicians and donors), organizational and other health system reform (e.g. decentralization in Mozambique and health insurance in Ghana) and, no doubt, socio-political change (perhaps, including in patient and political expectations of the health system).

**Table 1:** Features of complex social programs

• Based on set of theories and assumptions about how an intervention will lead to change
• Achieved through active participation of individuals

• Developed and implemented through long process which may be fallible

• Not necessarily implemented in linear fashion and influenced by respective power of those actors involved in implementation

• Very susceptible to effect of different contexts (e.g. policy timing, organizational culture and leadership, resource allocation, staffing levels and capabilities, interpersonal relationships, and competing local priorities and influences)

• Prone to being changed during process of implementation

• Open dynamic systems in themselves, which are able to change the conditions that enable them to be implemented successfully (generating unintended positive and negative effects)

Source: Mills et al, 2008 [5], adapted from Pawson et al, 2005 [13]

There is much to learn from these experiences, and evaluation is a central element of the AHI. Against the backdrop of the increased resources for the “big diseases” achieved in the early 2000s, this evaluation seeks to show whether or not investment in health systems and at scale, rather than in particular strategies for managing responsive primary care conditions, can generate health gain [4]. The effort and time put into the evaluation itself signals the importance of learning from these experiences and will also generate methodological lessons.

However, fully capturing the AHI’s lessons about implementing innovative health system development activities will also require other evaluation approaches. At present, the primary evaluation question being asked is, in essence: Can multi-dimensional health system strengthening initiatives offer health gain? This question is of particular significance in international health debates, and to funding agencies. But it does not fully address the concerns of those working within health systems and responsible for their continuous improvement. Over time, they have to manage variable investment levels and patterns, as well as changing political imperatives, demand patterns, health needs, and other system shocks. Health system managers are more likely to ask questions, such as what changes in the health system did these initiatives bring about, and how? Were there unintended consequences and, if so, how were they managed? What possibilities did the initiatives create for supporting forward momentum towards long-term health and development goals? Close examination of implementation experience is also important in addressing issues, such as “whether the promise of performance gains in well-resourced pilot studies can be achieved more widely; the replicability of experience across different contexts; the management strategies that can support effective implementation; and why change generates unexpected and unwanted effects” [5].

The existing knowledge base provides ideas of the sorts of issues likely to influence implementation within the PHIT Partnerships. For example, wider experience of health policy implementation [6] highlights that it:

• often results in unintended and unwanted consequences;

• is always contested by policy actors — not just politicians or interest groups contesting political agendas, but also, and as importantly, those actors working within the implementation chain, such as managers, health workers, patients, civil society organizations;

• is strongly influenced by the meanings policy actors attribute to features of design or to policy goals, which influences how they understand them and then react to them.

The implications are that managing implementation requires deliberate engagement with the values, interests, and understandings of those actors who might block or subvert policy change.

Experience of scaling up innovative public health programs also provides insights into the sorts of issues and management factors that influence the implementation of new health system initiatives. In a Kenyan program in which private shopkeepers were trained to provide malaria treatment, successful scaling up was supported by local level action and learning, combined with management strategies that were responsive to unexpected events and addressed tensions among implementing actors. The provision of technical support and adequate resources were, therefore, judged as vital, but not sufficient on their own to support scale-up [7]. Reflection on the South African experience in sustaining large scale antiretroviral scale-up, meanwhile, points to the importance of leadership, a combination of program standardization and flexibility, “clinical” partnerships, and monitoring and evaluation systems [8]. Broader innovation literature [9] finally, suggests that in order to institutionalize innovations within health systems it is essential to reorient existing organizational norms, values, incentives, and traditions in ways that encourage implementing actors to support new ways of working.

Implementing change within health systems must, therefore, work across both dimensions of the “whole system”. Evaluation of implementation requires strategies that take account of that complexity. This is the aim of “theory driven inquiry” [10]. Such inquiry moves beyond outlining the basic program theory of an intervention, as reflected in the PHIT Partnership descriptions presented here. It seeks, in addition, to understand how an intervention - the management of its implementation, or the way it plays out in a specific context - influences key actors to behave in ways that support (or work against) the innovation’s implementation. Identifying these trigger mechanisms, and the underlying assumptions about how and why they generate the expected behavioral changes, is the central focus of evaluation. In theory driven inquiry, these ideas are developed and examined through the process of evaluation and across several case studies of intervention, and are revised and refined in response to empirical experience. The primary question of focus in such evaluation is “what works for whom and in what circumstances”?

A burgeoning range of development and social policy literature provides guidance about how to develop these theories of change [11]. Drawing specifically on four key sets of questions [12] could provide the starting point for understanding the experience of each PHIT Partnership. These questions are:

• What overall health system change/situation does it seek to achieve? (that is plausible in itself and as a step to health gain) What features of this change/situation are the focus of partnership activities?

• Who are the agents of change? Which actors have to be involved to support health system change? What position and interests do they hold in relation to the change(s) envisaged? How does that influence their response to it?

• What are the expected mechanisms and pathways of change? What behavioral drivers are embedded in the intervention?

• How does the partnership team (the support or resource team) work (with whom) to support the intervention directly and to provide a supportive context for the intervention (e.g., through feedback higher up the system)?

Investigating and understanding the assumptions underlying each partnership’s initial plans and how these changed in response to experience would also be an essential part of such evaluation.

The full learning potential of the AHI lies in considering all dimensions of these health system/whole system changes. Evaluation must move beyond the “did it work” question, to consider why “it” worked, in what ways, and for whom, and, equally as importantly, why “it” didn’t work in other dimensions, and for whom. Such knowledge will add to our understanding of what shapes and filters health system functioning and performance, as well as offering insights about how to support future health system development.

## List of abbreviations used

AHI: African Health Initiative; PHIT: Population Health Implementation and Training

## Competing interests

The author declares that she has no competing interests.

## Declarations

This article has been published as part of *BMC Health Services Research* Volume 13 Supplement 2, 2013: Improving primary health care to achieve population impact: the African Health Initiative. The full contents of the supplement are available online at http://www.biomedcentral.com/bmchealthservres/supplements/13/S2. Publication of this supplement was supported by the African Health Initiative of the Doris Duke Charitable Foundation.
